# Reduced Endoplasmic Reticulum Stress-Mediated Autophagy Is Required for Leptin Alleviating Inflammation in Adipose Tissue

**DOI:** 10.3389/fimmu.2017.01507

**Published:** 2017-11-08

**Authors:** Lu Gan, Zhenjiang Liu, Dan Luo, Qian Ren, Hua Wu, Changxing Li, Chao Sun

**Affiliations:** ^1^College of Animal Science and Technology, Northwest A&F University, Yangling, China

**Keywords:** leptin, endoplasmic reticulum stress, autophagy, Atf4/Atg5, inflammation, adipocyte

## Abstract

Leptin is an adipocyte-derived hormone and maintains adipose function under challenged conditions. Autophagy is also essential to maintain cellular homeostasis and regulate characteristics of adipose tissue. However, the effects of leptin on autophagy of adipocyte remain elusive. Here, we demonstrated endoplasmic reticulum (ER) stress and leptin were correlated with autophagy and inflammation by transcriptome sequencing of adipose tissue. Leptin-mediated inhibition of autophagy was involved in upstream reduction of ER stress proteins such as Chop, GRP78, and Atf4, since blockage of autophagy using pharmacological approach had no effect on tunicamycin-induced ER stress. Moreover, we determined *KLF4*, the potential transcriptional factor of *Atf4*, was required for the leptin-mediated autophagy in the regulation of adipocyte inflammation. Importantly, ATF4 physically interacted with ATG5 and subsequently formed a complex to promote adipocyte autophagy. Further analysis revealed that Atg5, a core component of autophagosome, was the target for leptin-mediate autophagy. In addition, leptin alleviated ER stress-induced inflammation by reducing autophagy-mediated degradation of IκB in adipocytes. Exogenous leptin treatment also ameliorated autophagy and inflammation of white adipose tissue in *ob/ob* mice. Taken together, our results indicated that leptin inhibited ER stress-mediated autophagy and inflammation through the negatively regulation of Atf4/Atg5 complex in adipocytes. These findings identify a new potential means for intervention of autophagy to prevent or treat obese caused metabolic syndrome of mammals.

## Introduction

Metabolic disorders, especially obesity and diabetes, become the most important public health concern in worldwide. The pathophysiology seems to be largely attributable to endoplasmic reticulum (ER) stress, inflammation and cytokines resistance (e.g., leptin resistance and insulin resistance) in adipose tissue. Therefore, dynamic improvement of these disorders in adipose tissue provides an effective therapeutic strategy to alleviate severe systemic metabolic disorders.

Leptin is a hormone produced by peripheral adipose tissue. The ectopic serum leptin level is usually considered as a symbol of metabolic disorders (1–3). In particular, leptin signal is essential to mediate protective function in adipocytes under challenged conditions (1, 4, 5). Meanwhile, obesity is usually associated with mild chronic inflammation in adipose tissue. Notably, inflammatory stimulation by tumor necrosis factor-alpha (Tnfα) and other cytokines initiate adipose inflammation through elevating unfolded protein response in the ER (6, 7). ER plays a critical role in protein synthesis, folding and transportation. Certain conditions, such as accumulation of misfolded proteins and uncontrolled energy homeostasis perturb ER homeostasis and lead to a condition defined as ER stress (8–10). Recent studies show that ER stress serves as a key etiologic component causing chronic inflammation in adipose tissue of obese human and mice (11, 12). Current evidence suggests that ER stress promotes inflammation through positive transcription regulation of inflammatory genes and coordinating the activities of critical inflammatory kinases such as nuclear factor-κB (NF-κB), c-Jun N-terminal kinase, and protein kinase R (13–15). Our previous study has shown that tunicamycin (TM) induces ER stress and reduces adiponectin by increasing the *activating transcription factor 2* (*Atf2*) transcription in mice adipose tissue (12). Since leptin is an adipocyte-derived hormone involved in obesity and inflammation (5, 16), the specific role of leptin in the progress of ER stress-induced adipose inflammation remains to be determined.

Autophagy is a highly regulated process that functioned in the maintenance of cellular homeostasis (17, 18). Recently, studies indicate activation of ER stress could initiate autophagy and inflammation in astrocytes (19, 20). Many studies also determine that ER stress triggers autophagy and subsequent lysosomal lipolysis in hepatocytes (21, 22). Furthermore, autophagy can directly degrade lipid droplets through hydrolysis of triglycerides into free fatty acids in adipose tissue (23, 24). But the relationship between ER stress and autophagy in adipocytes remains elusive. Generally, the autophagy-related genes (Atg) recruit damaged organelles to form autophagosomes and recycle the cargo by lysosomal degradation (23, 25, 26). Deletion of autophagy-related gene 7 (Atg7) blocks autophagy and promotes hepatic lipid accumulation (27). Adipocyte-specific deletion of Atg genes exhibit markedly decreased plasma concentration of leptin (28, 29). These findings suggest leptin may be correlated with autophagy by regulating ER stress in adipocytes.

In this study, we investigated the effects of leptin on autophagy and inflammation of adipose tissue. We show that leptin alleviates ER stress-induced inflammation through the activating transcription factor 4 (Atf4)/Atg5-mediated autophagy in mice adipocytes. Thus, our study has revealed the mechanistic link between leptin, autophagy and ER stress, providing novel insights into the pharmacologically therapeutic target for obesity and inflammation.

## Materials and Methods

### Animal Experiment

Eight-week-old C57BL/6J male mice and *ob/ob* mice were purchased from the Laboratory Animal Center of the Fourth Military Medical University (Xi’an, China). Mice handling protocols were conducted following the guidelines and regulations approved by the Animal Ethics Committee of Northwest A&F University (Yangling, China). Mice were provided *ad libitum* water and a standard laboratory chow diet purchased from Animal Center of the Fourth Military Medical University. Body weight was recorded weekly. The animal room was maintained at 25 ± 1°C, humidity at 55 ± 5%, and 12 h light/dark cycles.

Mice (*n* = 24) were randomly divided into four groups (*n* = 6 each). Half of the mice were intraperitoneally injected with saline (control), and the other half injected with TM (1 mg/kg, Sigma-Aldrich, MO, USA, T7765) or thapsigargin (Tg, 0.5 mg/kg, Sigma-Aldrich, MO, USA, T9033) for 3 and 2 days separately before the dark onset (12, 30–32). The TM injection was used to create ER stress in the mice adipose tissue. To address the effect of leptin on ER stress of adipose tissue, half of the mice that received saline or TM or Tg injection were further injected with 1 mg/kg recombinant murine leptin (Peprotech, NJ, USA, 450-31) into the tail vein two times a day for 7 days (15, 33, 34), and the other half still received saline injection. For the experiment of 4-phenylbutyric acid (4-PBA, 0.5 g/kg, Sigma-Aldrich, MO, USA, P21005) injection (35), mice (*n* = 24) were all injected with TM (1 mg/kg) and randomly divided into four groups (*n* = 6 each). Half of these mice received saline injection (control), and the other half received 4-PBA injection (0.5 g/kg) for 7 days. And then half of the mice that received 4-PBA or saline injection were injected with recombinant murine leptin (1 mg/kg) two times a day for 7 days. For the rapamycin (5 mg/kg, Sigma-Aldrich, MO, USA, V900930) injection experiment (36), we performed the same procedures as in the 4-PBA injection experiment. 4-PBA injection was used to block ER stress and rapamycin injection was used to activate autophagy. In the *ob/ob* mice experiment (*n* = 18), mice were randomly divided into three groups (*n* = 6 each group), one group of mice was the C57BL/6J mice [wild type (WT)], the other two groups were *ob/ob* mice. Half of the *ob/ob* mice were injected with saline (control) and the other half injected with 1 mg/kg recombinant murine leptin (1 mg/kg) two times a day for 7 days. Mice were then euthanized by ethyl ether. The epididymal white adipose tissue (eWAT) was dissected and was used for the following studies. Serum leptin level was measured by the commercial enzyme-linked immunosorbent assay (ELISA) kit from Sigma (Sigma-Aldrich, MO, USA, RAB0334,), and the measurement kits of interleukin (IL)-18 and IL-1β were from Abcam (UK, ab216165, ab100704) following the manufacturer’s instructions and our previously study (37).

### Primary Adipocyte Culture and Virus Vectors Infection

The connective fiber and blood vessels of eWAT tissues were removed, and washed three times with PBS buffer containing penicillin and streptomycin (Sigma-Aldrich, MO, USA, P3032, S6501). The adipocyte culture was carried out according to our previous publication (38). Briefly, adipocytes were seeded onto 35-mm culture dishes at 30% (v/v) confluence, and incubated at 37°C under a humidified atmosphere of 5% CO_2_ and 95% air for subsequent experiments. After reaching 95% confluence, preadipocytes were induced to differentiate using DMEM/F12 (Gibco, CA, USA, 12500062) with 15% FBS and 100 nM insulin for 5–6 days until exhibiting a massive accumulation of fat droplets. Leptin (Peprotech, NJ, USA, 450-31) was added into culture medium at a final concentration of 100 ng/mL and incubated for up to 24 h. Adipocytes were preincubated with 3-methyladenine (3-MA, 5 mM for 2 h, Selleck, China, S2767) or 4-phenylbutyrate (4-PBA; 50 mM for 1 h, Sigma-Aldrich, MO, USA, SM0309). Then followed treated with leptin for 12 h or 16 h separately, then TM (2 µg/mL, Sigma-Aldrich, MO, USA) or rapamycin (100 nM, Selleck, China, S1039) were further added into the culture medium for 12 or 8 h, respectively. For autophgic flux detection, adipocytes were exposed to leptin or TM followed by treatment of cells with an inhibitor of autophagosome-lysosome fusion, bafilomycin A1 (BafA1) (400 nM in the last 4 h of the 24 h treatment period), and assessed for accumulation of LC3II. For vectors infection study, adipocytes were infected with overexpression adenovirus or interference lentiviral recombinant vectors of *leptin* (pAd-*Leptin* or si-*Leptin*), *Atg5* (pAd-*Atg5* or si-*Atg5*), or Krüppel-like factor 4 (*KLF4*, pAd-*KLF4* or si-*KLF4*) for 48 h at the titer of 1 × 10^9^ IFU/mL, and then treated with leptin. The control vectors were pAd-GFP or pGLVU6-GFP. All the vectors were constructed by Gene Pharma (China).

### Detection of Autophagy Incidence by Flow Cytometry (FACS)

After treatment with different conditions, the adipocytes were incubated with 0.05 mM monodansylcadaverine (MDC, Sigma-Aldrich, MO, USA, 30432) at 37°C for 30 min and then washed three times with PBS. Intracellular MDC was measured by flow cytometry (FACS) within 30 min after incubation. Fluorescence intensity of cells was determined by BD FACScan (BD Biosciences, NJ, USA) and data were analyzed using Cell Quest software (BD Biosciences).

### GFP-LC3 Analysis and Subcellular Localization

Cells were transfected with GFP-LC3 plasmid by using X-treme GENE HP Reagent (Roche, Switzerland) according to the manufacturer’s instructions. After 48 h transfection, cells were washed with OptiMEM I (Invitrogen, CA, USA, 51985042) and subjected to staining. The cells were stained with LysoTracker^®^ Green DND probe (Thermo Scientific, CA, USA, L7526) as recommended by the manufacturer. The formation of GFP-LC3 punctate and Tracker fluorescence were visualized and analyzed using Cytation3 Cell Imaging Multi-Mode Reader (BioTek, VT, USA).

### Transmission Electron Microscopy (TEM)

At room temperature eWAT or adipocytes were fixed in 2.5% glutaraldehyde in PBS (pH = 7.2) for 24 h, postfixed in 1% osmium tetroxide in water for 2 h. After dehydrated in an ascending series of ethanol (30, 50, 70, 80, 90, and 100%) for 10 min each, the samples were then embedded in Durcopan ACM (Fluka Chemie AG, Switzerland, 44611). Sections were cut with a diamond knife at a thickness of 50–60 nm. These sections were stained with uranyl acetate and lead citrate, and examined with a TEM (HT7700, 80 kV, Hitachi, Japan). Images were recorded on film at 30,000× magnification. The percentage of mitochondrial integrity was determined by dividing the number of normal mitochondria by the total number of mitochondria per image.

### RNA-Seq Analysis

Total RNA from the eWAT was prepared with RNAiso Reagent (Takara, China, D312) and the RNA-seq analysis was performed as previous described (39). Briefly, quantification and quality control of the sample libraries were assessed by Agilent 2100 Bioanalyzer and ABI StepOnePlus real-time PCR system. RNA sequencing was performed using Hiseq 4000 instrument (Illumina). Real-time analysis was used for base calling. Fastq files were mapped to the mouse genome (NCBI37/mm9) using TopHat (version 2.0.4). Mapped reads were then assembled *via* Cufflinks (version 2.0.2) with the default settings. Assembled transcripts were then merged using the Cuffmerge program with the reference genome. Analysis of mRNA levels was carried out using the Cuffdiff program, with samples being grouped by treatment condition, three replicates per group. Volcano plots comparing log10 (statistical relevance) to log2 (fold change) were generated using R (version 3.1.1), using the base plotting system and calibrate library. Gene Ontology (GO) and pathway enrichment analysis were performed to categorize the considerably enriched functional classification or metabolic pathways in which DEGs operated.

### Plasmids Construction and Dual-Luciferase Reporter Assay

A 1,267 bp mouse *Atf4* promoter was cloned by PCR amplification of C57BL/6J mouse genomic DNA and inserted in the pGL-3 basic vector. The resulting reporter was named *Atf4*_1267_-Luc. Further deletion of the *Atf4*_1267_-Luc generated *Atf4*_720_-Luc, *Atf4*_560_-Luc and *Atf4*_130_-Luc reporters contained of 720, 560, and 130 bp of *Atf4* promoter, respectively. HEK293 cells were cotransfected with luciferase reporter plasmid, pRL-TK reporter plasmid (control reporter), and KLF4 plasmid (pc-*KLF4*) using X-tremeGENE™ transfection reagent (Roche, Switzerland, 06366236001). After transfection for 24 h, cells were harvested and measured using the dual-luciferase reporter assay system (Promega, WI, USA, E1910), and luciferase activity was divided by the Renilla luciferase activity to normalize for transfection efficiency.

### Chromatin Immunoprecipitation (ChIP) Assay

Chromatin immunoprecipitation assay was performed as previous described using a ChIP assay kit (Abcam, UK, ab500) according to the manufacturer’s protocol (12). Primary antibodies of Atf4 and IgG (Abcam, UK, ab172730) were used. DNA–protein crosslinking complexes were collected, and purified DNA was subjected to qPCR with SYBR green fluorescent dye (Invitrogen, CA, USA).

### Coimmunoprecipitation (Co-IP) Analysis

HEK293 cells were transfected with plasmids using X-tremeGEN™ transfection reagent (Roche, Switzerland, 06366236001) as previous described (32). After 24 h transfection, cells were then snap-frozen in lipid nitrogen. Whole cell lysate was harvested in lysis buffer with a protease inhibitor. Cells were then sonicated for 10 s and the whole cell lysate was precleared with Protein A for 2 h and incubated with 2 µg primary antibody overnight at 4°C. Immune complexes were pulled down with Protein A agarose for 2 h at 4°C with shaking. Beads were washed once with lysis buffer and three times with wash buffer, and then eluted by boiling in SDS sample buffer followed by detection of Western blot.

### Nuclear Protein Extraction

Nuclear and cytoplasmic fractions were prepared using the protocols from Liu et al (40). In brief, cells were lysed with 400 µL of cytoplasmic lysis buffer. The lysates were incubated for 5 min on ice and vortexed 2 times for 10 s. The lysates were centrifuged for 30 s at 16,000*g*, and supernatants were collected as cytoplasmic fractions. The pellets were re-suspended in 50 µL of nuclear extraction buffer and sonicated 3 times on ice. The nuclear fractions were centrifuged for 5 min at 16,000*g*, and the supernatant was collected to obtain nuclear proteins. The proteins were denatured by boiling at 100°C and kept for further studies.

### Real-time Quantitative PCR Analysis

Total RNA of eWAT or adipocytes were extracted with TRIpure Reagent kit (Takara, China) according to previous study (41). 500 ng of total RNA was reverse transcribed using M-MLV reverse transcriptase kit (Invitrogen, USA, 28025013). Primers were synthesized by Invitrogen (China). Quantitative PCR was performed in 25 µL reaction system containing specific primers and AceQ qPCR SYBR Green Master Mix (Vazyme, China, Q111-02). Amplification was performed in the ABI StepOne Plus™ RT-PCR System (CA, USA). The levels of mRNA were normalized in relevance to GAPDH. The expression of genes was analyzed by method of 2^−ΔΔCt^.

### Protein Extraction and Western Blot Analysis

Western blot was performed as previously described (31). Adipocytes were solubilized in lysing buffer. Protein samples (30 µg) were separated by electrophoresis on 12 and 5% SDS-PAGE gels using slab gel apparatus, and transferred to PVDF nitrocellulose membranes (Millipore, USA). Antibodies including LC3II (ab48394), Atg5 (ab108327), Beclin1 (ab62557), SQSTM1 (ab51416), signal transducer and activator of transcription 3 (STAT3, ab68153), p-STAT3 (ab76315), JAK2 (ab108596), p-JAK2 (ab32101), Atf4 (ab184909), glucose-regulated protein 78 (GRP78, ab21685), C/EBP homologous protein (Chop, ab11419), p65 (ab16502), IκB (ab32518), p-IκB (ab92700), NLRP3 (ab214185), IL-18 (ab71495), PCNA (ab29), GAPDH (ab8245), anti-HA tag (ab18181), and anti-His tag (ab18184) were purchased form Abcam (UK) and IL-1β (12426) from cell signaling technology (USA), the appropriate HRP-conjugated secondary antibody (Boaoshen, China) were used. Proteins were visualized using chemiluminescent peroxidase substrate (Millipore, USA), and then the blots were quantified using ChemiDoc XRS system (Bio-Rad, USA).

### Statistical Analysis

Statistical analyses were conducted using SAS v8.0 (SAS Institute, NC, USA). Data were analyzed using one-way or two-way ANOVA. Comparisons among individual means were made by Fisher’s least significant difference. In consideration of multiple testing, the significance level was corrected using the Bonferroni method. Data were presented as mean ± SEM. *p* < 0.05 was considered to be significant.

## Results

### Leptin Produces a Transcriptional Signature Distinct from TM and Reduces Genes Associated With Autophagy and ER Stress in Mice Adipose

**Figure 1:**
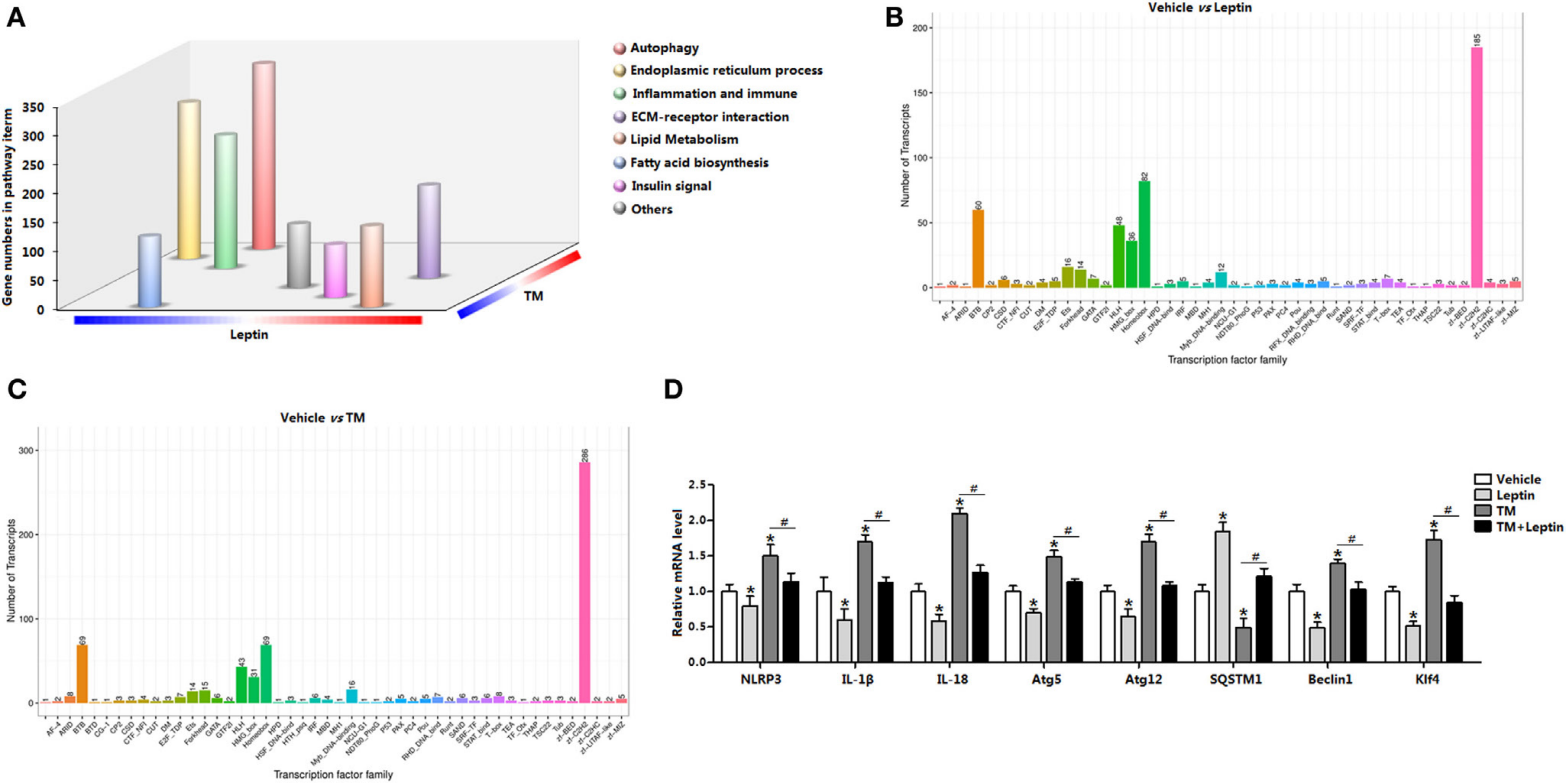
Leptin treatment produces a transcriptional signature distinct from tunicamycin treatment and is defined by the induction genes associated with autophagy and endoplasmic reticulum (ER) stress in mice adipose. **(A)** Gene Ontology (GO) analysis of the genes set from RNA-seq data (*n* = 3). **(B)** Transcription factor family in genome after leptin treatment (*n* = 3). **(C)** Transcription factor family in genome after TM treatment (*n* = 3). **(D)** Changes in the expression level of genes associated with autophagy and inflammation, which were significantly altered from RNA-seq analysis (*n* = 3). Values are means ± SEM. *p < 0.05 compared with the vehicle group, ^#^*p* < 0.05 compared with the tunicamycin (TM) group, ^&^*p* < 0.05 compared with the leptin group.

We used RNA-seq and functional enrichment analysis to compared white adipose transcriptomes from leptin and TM-injected mice. Notably, a total of 12,732 genes were found to be significantly altered, and 68% (8,650 out of 12,732) of the genes were increased in leptin-injected mice, whereas 32% (4,081 out of 12,732) were decreased (Figure S1A in Supplementary Material). Similarly, TM injection altered 12,588 genes expression, and 62% (7,767 out of 12,588) of the genes were increased, whereas 38% (4,821 out of 12,588) were decreased (Figure S1A in Supplementary Material). GO analysis showed 22% of these genes were mainly functioned in signal transduction, 18% functioned in protein digestion, and 14% functioned in inflammation response (Figure S1B in Supplementary Material). Subsequent analysis showed leptin downregulation of 4,081 genes and TM upregulation of 7,767 genes contemporarily, and the genes were enriched in those encoding factors involved in autophagy (Figure S1C in Supplementary Material), suggesting leptin and TM treatment resulted in a regulation of autophagy. Further pathway analysis of the TM and leptin coregulated genes revealed that autophagy, ER stress and inflammation pathways were all highly enriched (Figure 1A), demonstrating that genes regulated by TM and leptin were correlated with autophagy and inflammation processes in adipose tissue. The data also showed the transcription factor cluster of multiply genes were altered, suggesting the existence of transcription regulation between these genes (Figures 1B,C). mRNA expression measurement established that autophagy marker genes *autophagy-related gene 5* (*Atg5*), *autophagy-related gene 12* (*Atg12*), and *Beclin1* were decreased in leptin group but increased in TM group; while the cotreatment of leptin and TM reduced these genes expression compared with that in TM alone group (Figure 1D). Inflammation indicators *IL-18, IL-1β*, and *NLR family, pyrin domain containing 3* (*NLRP3)* were also decreased after leptin treatment, but increased with TM injection; cotreatment of TM and leptin further decreased the levels of these genes (Figure 1D). These results were also confirmed with another ER stress inducer, the Tg treatment (Figures S2B,C in Supplementary Material). These findings indicated ER stress accelerated autophagy, but leptin played the opposite role in the regulation of both ER stress and autophagy in adipose tissue.

### Leptin Reduces ER Stress-Induced Autophagy and Alleviates Inflammation Cytokines Secretion in Mice Adipose Tissue

**Figure 2:**
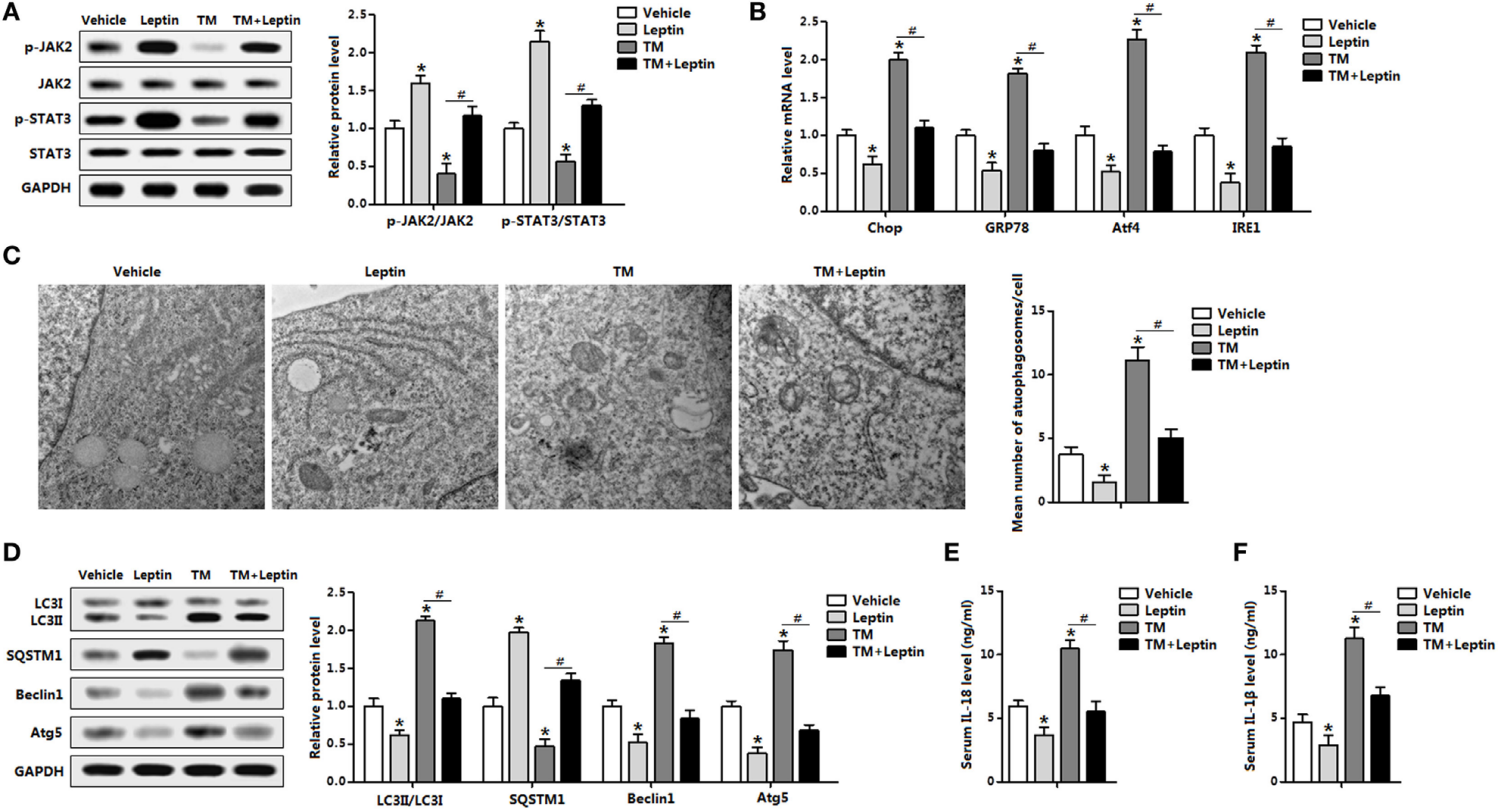
Leptin treatment reduces endoplasmic reticulum (ER) stress-induced autophagy and alleviates inflammation cytokines secretion in mice adipose tissue. Mice were injected with tunicamycin (TM) or leptin, the white epididymal adipose tissue (eWAT) or serum was used for this study (*n* = 6). **(A)** Leptin sensitivity markers [phosphorylation of Janus kinase 2 (JAK2) and signal transducer and activator of transcription 3 (STAT3)] were examined by Western blot analysis. **(B)** Gene expression levels of ER stress markers: C/EBP homologous protein (*Chop*), glucose-regulated protein 78 (*GRP78*), activating transcription factor 4 (*Atf4*), and inositol-requiring enzyme 1 (*IRE1*). **(C)** Representative electron micrographs (30,000×) of eWAT treated with TM or leptin. The graph showed on the right was the quantification of mean number of autophagosomes in the EM images. **(D)** Autophagy was evaluated by the conversion of LC3I to LC3II, the protein levels of Beclin1, Atg5, and SQSTM1 were detected by Western blot analysis. **(E)** Serum interleukin (IL)-18 level measured by enzyme-linked immunosorbent assay (ELISA) test. **(F)** Serum IL-1β level measured by ELISA test. Full scans of uncropped blots are included in Figure S3. Values are means ± SEM. *p < 0.05, ^#^*p* < 0.05 compared with the TM group.

The distinct responses of leptin and ER stress to autophagy were further demonstrated with the cotreatment of leptin and TM *in vivo*. Leptin markedly enhanced the phosphorylation levels of STAT3 and kinase 2 (JAK2) which were blunted by TM injection, indicating leptin signal was sensitive to leptin and TM treatments (Figure 2A). As expected, TM treatment triggered adipose ER stress and the addition of leptin attenuated the elevation of *Chop, GRP78, Atf4*, and *inositol-requiring enzyme 1* (*IRE1*) (Figure 2B). And Tg treatment also showed the consistent results (Figure S2A in Supplementary Material). As shown in Figure 2C, protein levels of classic autophagic markers LC3II, Beclin1, and Atg5 were significant induced by TM incubation whereas the level of SQSTM1 protein, a marker for autophagic flux, showed a decrease with TM treatment, suggesting the induction of autophagy by TM-induced ER stress. However, the addition of leptin showed the opposite results, indicating the reduction of autophagy (Figure 2D). Analyzing of autophagosomal structures of adipose tissue by TEM demonstrated autophagosome formation was increased in TM injected mice and decreased with leptin treatment, and changed in the cotreatment group, implying leptin reduced the autophagosome formation (Figure 2C). We also examine inflammation status with the cotreatment of leptin and TM. TM-induced ER stress promoted serum secretion of IL-18 and IL-1β, while the addition of leptin had the opposite effect (Figures 2E,F). Taken together, these observations indicated leptin treatment reduced ER stress-induced autophagy and alleviated inflammatory cytokines secretion of mice adipose tissue.

### Leptin Decreases ER Stress-Induced Autophagy Flux by Reducing LC3II Turnover and SQSTM1 Degradation in Mice Adipocytes

**Figure 3:**
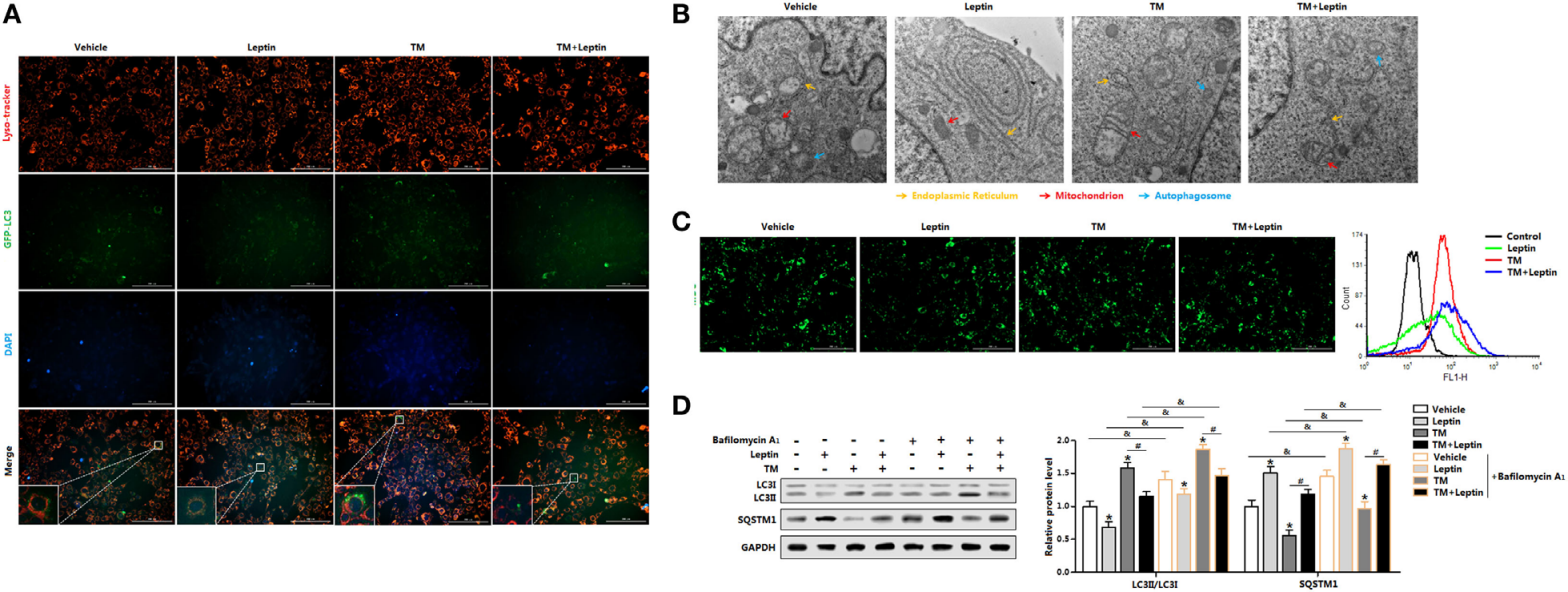
Leptin decreases endoplasmic reticulum (ER) stress-induced autophagy flux by reducing LC3II turnover and SQSTM1 degradation in mice adipocytes. **(A)** Representative fluorescent photomicrographs showing the GFP-LC3 puncta formation in adipocytes transfected with GFP-LC3 plasmid and stained with Lyso-tracker Red. Cells were treated with tunicamycin (TM) or leptin. The nuclei were stained with DAPI shown in blue (*n* = 3). **(B)** Representative electron micrographs (30,000×) of adipocytes. Yellow arrowhead: endoplasmic reticulum, red arrowhead: mitochondria, blue arrowhead: autophagosome (*n* = 3). **(C)** Representative pictures of autophagosome formation monitored by MDC staining. Cell autophagy was analyzed by flow cytometry (*n* = 3). **(D)** Representative Western blots showing the protein levels of LC3II and SQSTM1 in adipocytes treated with leptin or TM followed by treatment with 400 nM bafilomycin A1 (BafA1), which was added in the last 4 h of the treatment period (*n* = 3). Full scans of uncropped blots are included in Figure S3. Values are means ± SEM. *p < 0.05 compared with the control group, ^#^ *p* < 0.05 compared with the TM group, ^&^ *p* < 0.05 compared with the BafA1 group.

Having shown leptin was capable of reducing ER stress-induced autophagy in mice adipose tissue, we next sought to confirm this by measuring the autophagosome formation in adipocytes *in vitro*. We first transfected adipocytes with overexpression GFP-LC3 plasmid followed incubated cells with TM or leptin, and subsequently detected the green fluorescent LC3II puncta. TM exposure prominently triggered the formation of characteristic punctate GFP-LC3, suggesting the recruitment of GFP-LC3 during autophagosome formation (Figure 3A). Cotreatment of TM and leptin resulted in the decrease in the percentage of cells with punctate GFP-LC3 compared with the cells treated with TM alone (Figure 3A), confirmed the reduction of autophagosome formation by leptin. We then analyzed the TEM pictures of adipocytes pretreated with TM or leptin. Notably, autophagosome formed in the TM and leptin cotreated cells were smaller than that in the TM exposure cells, and much more than that in leptin group (Figure 3B). These findings were further validated by the MDC staining and FACS measurement that leptin reduced the autophagosome formation of adipocytes (Figure 3C).

Since leptin decreased the number of autophagosome in adipocytes, it is needed to distinguish whether the reduction of autophagosome is due to the de-activation of autophagy or rather due to an enhanced of autophagosome-lysosomal fusion at later stages (21). To determine this, we exposed the cells to the lysosomal V-ATPase inhibitor BafA1, which blocks LC3II/autophagosome degradation and reveals changes in autophagosome synthesis (21). Autophagic flux was measured through LC3II turnover assay, by measuring LC3II degradation in adipocytes treated with TM or leptin, and followed exposed with or without BafA1. As shown in Figure 3D, exposure of adipocytes with TM followed by treatment with BafA1 resulted in significant increase of LC3II levels compared with BafA1 alone group; but the addition of leptin showed the opposite result. The decreased accumulation of LC3II protein indicated the reduced autophagic flux in cells exposed to leptin. We next assessed the degradation of SQSTM1 by autophagy to monitor the autophagic flux. It was evident that SQSTM1 level was decreased in TM-treated adipocytes, but increased in the TM and leptin cotreatment cells (Figure 3D). Further measurements demonstrated SQSTM1 protein level was significantly increased in TM and leptin cotreated adipocytes followed treated by BafA1, compared with cells without BafA1 (Figure 3D). These findings underpin the potential of leptin to downregulated autophgic flux. Therefore, reduced autophagic flux is responsible for SQSTM1 degradation in leptin-treated adipocytes.

### Leptin-Mediated Autophagy and Inflammation Involved Upstream Activation of ER Stress in Mice Adipocytes

**Figure 4:**
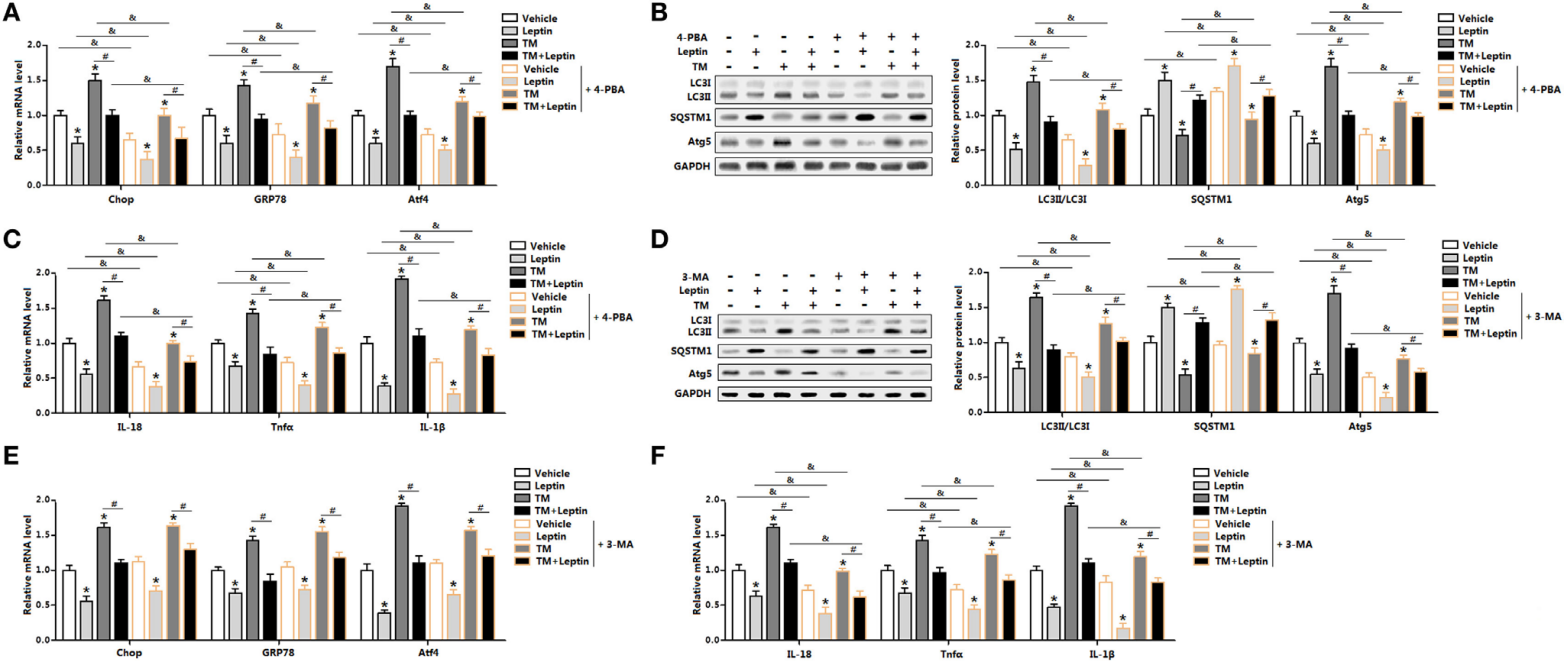
Leptin-mediated autophagy and inflammation involved upstream activation of endoplasmic reticulum (ER) stress in mice adipocytes. Adipocytes were pretreated with 4-phenylbutyric acid (4-PBA, 5 mM for 2 h) or 3-methyladenine (3-MA, 50 mM for 1 h), followed incubated with recombinant leptin for 24 h or tunicamycin (TM) for 12 h (*n* = 3). **(A)** Gene expression profile of C/EBP homologous protein (*Chop*), glucose-regulated protein 78 (*GRP78*), and activating transcription factor 4 (*Atf4*) in adipocytes treated with or without 4-PBA. **(B)** Representative Western blots showing the protein levels of LC3II, SQSTM1, and Atg5 in adipocytes treated with or without 4-PBA. **(C)** Gene expression profile of proinflammatory cytokines, such as *IL-18, Tnfα*, and *IL-1β* in adipocytes treated with or without 4-PBA. **(D)** Representative Western blots showing the protein levels of LC3II, SQSTM1, and Atg5 in adipocytes treated with or without 3-MA. **(E)** Gene expression profile of *Chop, GRP78*, and *Atf4* in adipocytes treated with or without 3-MA. **(F)** Gene expression profile of *IL-18, Tnfα*, and *IL-1β* in adipocytes treated with or without 3-MA. Full scans of uncropped blots are included in Figure S3. Values are means ± SEM. *p < 0.05 compared with the control group, ^#^*p* < 0.05 compared with the TM group, ^&^*p* < 0.05 compared with the 4-PBA group or 3-MA group.

We next determined the effect of ER stress inhibitor, 4-PBA, on leptin-mediated autophagy and adipocyte inflammation. As shown in Figure 4A, TM-induced ER stress was inhibited in cells with 4-PBA preincubation through the downregulation of *Chop, GRP78*, and *Atf4*. Compared with TM group, cotreatment of leptin and TM further strengthen the inhibition of ER stress by 4-PBA (Figure 4A) The protein levels of autophagy markers, LC3II and Atg5 were significantly decreased in TM and leptin cotreatment group compared with those in the control group without 4-PBA, but the level of SQSTM1 was increased (Figure 4B). Moreover, 4-PBA exposure led to the alleviation of adipocytes inflammation. As shown in Figure 4C, the mRNA levels of *IL-18, Tnfα*, and *IL-1β* were reduced significantly in leptin and TM cotreated cells which were pretreated with 4-PBA than that of alone cotreated cells.

Next, we explore the effect of 3-MA, a pharmacological autophagy inhibitor, on leptin-mediated autophagy and adipocytes inflammation. As presented in Figure 4D, protein levels of LC3II and Atg5 were inhibited in adipocytes incubated with 3-MA. Leptin-mediated the blockage of autophagy was further strengthened with the 3-MA treatment (Figure 4D). However, the mRNA expression profiles of ER stress markers, such as *Chop, GRP78*, and *Atf4* did not change significantly in leptin and TM cotreated cells preincubated with 3-MA than that of no 3-MA-treated cells (Figure 4E). Additionally, inflammatory markers were decreased in cells preincubated with 3-MA and followed treated with leptin and TM (Figure 4F). These findings confirmed that ER stress is the upstream for leptin-mediated autophagy and inflammation in mice adipocytes.

### *Leptin* Inhibited ER Stress-Induced Autophagy and Inflammation in Mice Adipocytes

**Figure 5:**
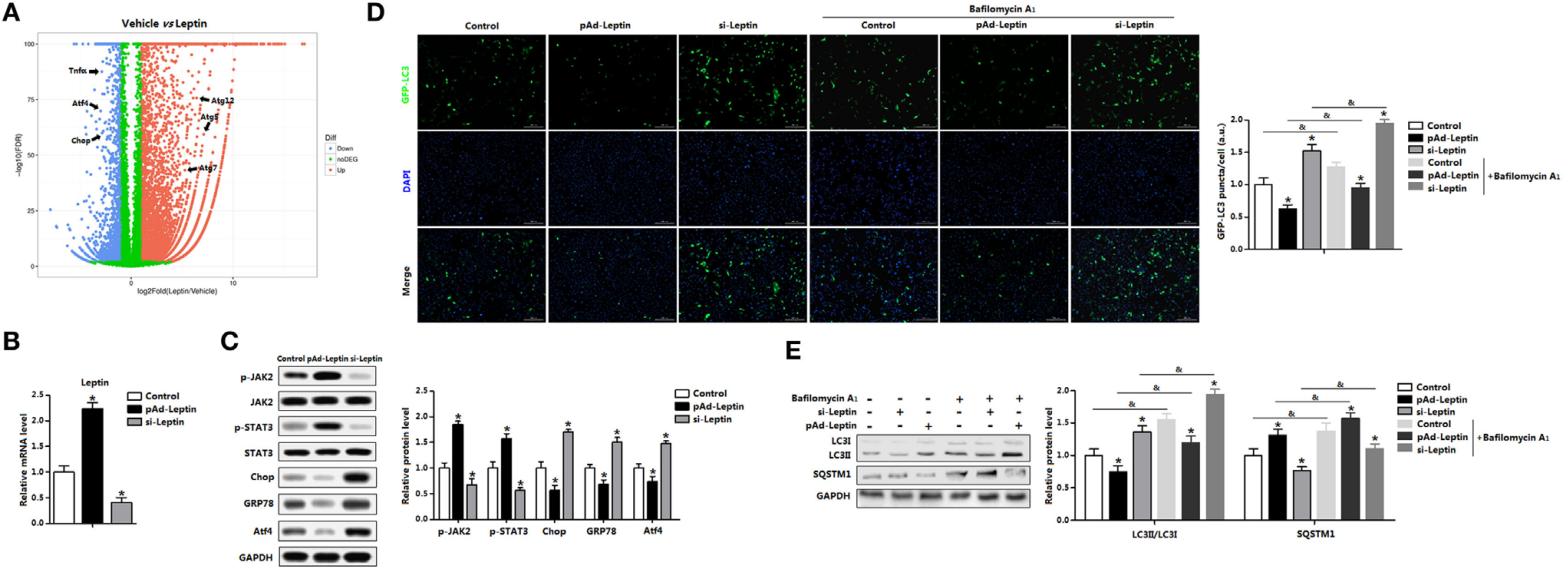
Overexpression of leptin inhibited autophagy in mice adipocytes. **(A)** Volcano plot of transcriptome in the white epididymal adipose tissue (eWAT) of mice injected with or without leptin (*n* = 3 each). Individual endoplasmic reticulum (ER) stress, autophagy, and inflammation signature genes were highlighted. Red: upregulated, blue: downregulated. **(B)** Gene expression level of *leptin* in adipocytes infected with pAd-*Leptin* or si-*Leptin* (*n* = 3). **(C)** Protein levels of phosphorylation of Janus kinase 2 (JAK2), signal transducer and activator of transcription 3 (STAT3), C/EBP homologous protein (Chop), glucose-regulated protein 78 (GRP78), and activating transcription factor 4 (Atf4) in adipocytes infected with pAd-*Leptin* or si-*Leptin* (*n* = 3). **(D)** Representative pictures of GFP-LC3 punctate structures in adipocytes expressing GFP-LC3, in the presence of pAd-*Leptin* or si-*Leptin* and pretreated with TM then incubated with or without bafilomycin A1 (BafA1). The nuclei were stained with DAPI shown in blue in adipocytes (*n* = 3). **(E)** Representative Western blots showing the protein levels of LC3II and SQSTM1 in adipocytes infected with pAd-*Leptin* or si-*Leptin* that pretreated with TM and followed treated with BafA1 or not (*n* = 3). Full scans of uncropped blots are included in Figure S3. Values are means ± SEM. *p < 0.05 compared with the control group, ^&^*p* < 0.05 compared with the BafA1 group.

We had demonstrated that signature genes regulated by leptin were associated with autophagy and inflammation from the volcano plot (Figure 5A). To confirm that leptin inhibited ER stress-induced autophagy and alleviated adipose inflammation, we forced expression or silenced of *leptin* (Figure 5B). Leptin signal sensitivity was significantly altered after leptin vectors infection as represented by the change of phosphorylation of JAK2 and STAT3 (Figure 5C). Consistently with the exogenous leptin treatment data, overexpression of *leptin* decreased the protein levels of Chop, GRP78, and Atf4 (Figure 5C). By contrast, interference of *leptin* observed the opposite results (Figure 5C). Next, we monitor autophagosome formation in adipocytes infected with pAd-*leptin* or si-*leptin*, the GFP-LC3 fusion protein was utilized. As expected, overexpression of *leptin* reduced the formation of exogenous GFP-LC3 puncta, and we found the consistent results in BafA1-treated group, indicating there was a decrease accumulation of autophagosome (Figure 5D). We next measured the autophgic flux *via* LC3II turnover assay and detected the degradation of SQSTM1. Cells infected with pAd-*leptin* followed by BafA1 resulted in a significant decrease of LC3II level. In addition, the evident upregulation of SQSTM1 in the present of pAd-*leptin* and BafA1 confirmed the decreased autophgic flux by leptin treatment in adipocytes (Figure 5E).

**Figure 6:**
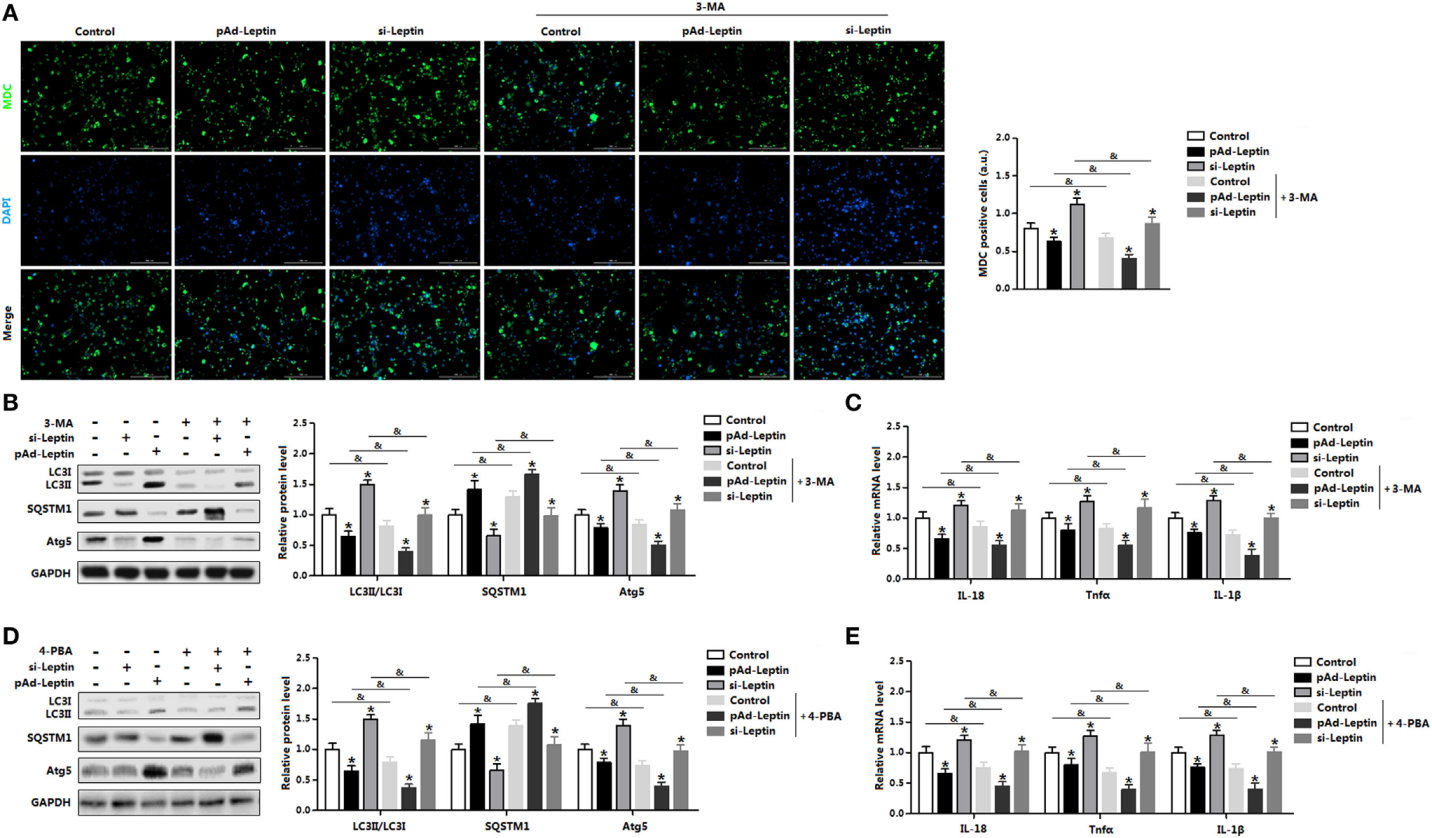
Leptin signal is essential for endoplasmic reticulum (ER) stress-induced autophagy and inflammation in mice adipocytes. Adipocytes were preinfected with pAd-*leptin* or si-*leptin* and incubated with tunicamycin (TM), then followed treated with 4-phenylbutyric acid (4-PBA, 5 mM for 2 h) or 3-methyladenine (3-MA, 50 mM for 1 h) (*n* = 3). **(A)** Representative pictures of autophagosome formation monitored by monodansylcadervarine (MDC) staining in adipocytes with or without 3-MA treatment. The nuclei were stained with DAPI shown in blue. **(B)** Representative Western blots showing the protein levels of LC3II, SQSTM1, and Atg5 in adipocytes treated with or without 3-MA. **(C)** Gene expressions of inflammation markers in adipocytes treated with or without 3-MA. **(D)** Representative Western blots showing the protein levels of LC3II, SQSTM1 and Atg5 in adipocytes treated with or without 4-PBA. **(E)** Gene expressions of inflammation markers in adipocytes treated with or without 4-PBA. Full scans of uncropped blots are included in Figure S3. Values are means ± SEM. *p < 0.05 compared with the control group, ^&^*p* < 0.05 compared with the 3-MA group or 4-PBA group.

We next determined the effect of 3-MA on leptin mediated autophagy. Monodansylcadervarine (MDC) staining indicated overexpression of *leptin* significantly reduced autophagy incidence in the cells preincubated with 3-MA (Figure 6A). The autophagy indicators LC3II and Atg5 were decreased with overexpression of *leptin* followed by 3-MA, while protein of SQSTM1 showed the opposite result (Figure 6B). Furthermore, *leptin* attenuated proinflammatory cytokines expression in cells pretreatment with 3-MA, similarly to the results showed in control group (Figure 6C). Overall, these findings implicated that leptin blocked ER stress-induced autophagy and alleviated inflammation in mice adipocytes.

### Leptin Reduces ER Stress *via* the Blockade of *Atf4* Transcription in Mice Adipocytes

**Figure 7:**
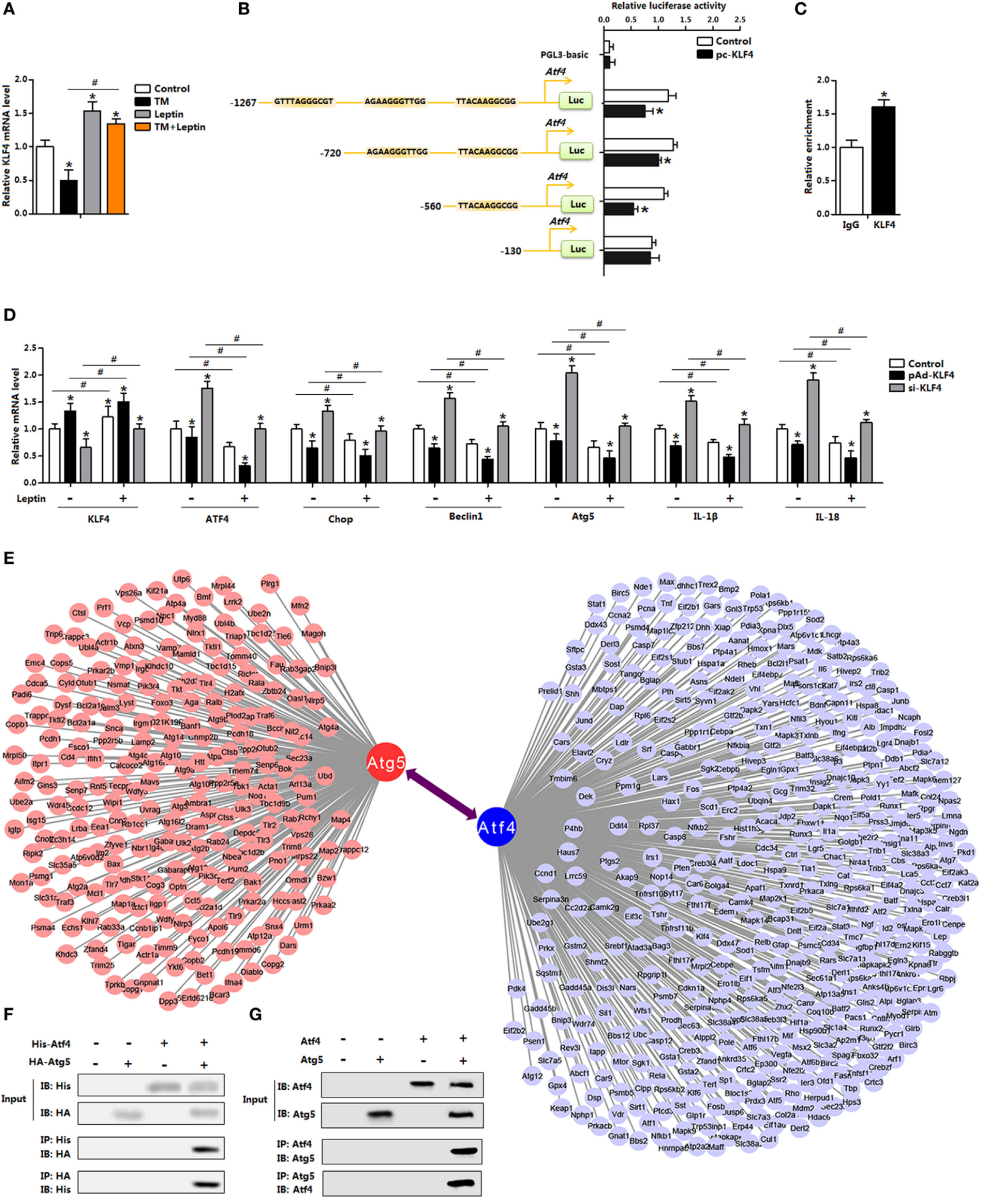
Leptin reduces endoplasmic reticulum (ER) stress *via* the blockade of activating transcription factor 4 (*Atf4*) transcription in mice adipocytes. **(A)** Gene expression of Krüppel-like factor 4 (*KLF4*) in adipocytes incubated with tunicamycin (TM) or leptin (*n* = 3). **(B)** Dual luciferase reporter assay of *KLF4* and *Atf4*. Cells were transfected with PGL3-basic or PGL3-*Atf4* plasmids, and pc-*KLF4* plasmid (*n* = 3). **(C)** Chromatin immunoprecipitation (ChIP) analysis between Atf4 and KLF4 (*n* = 3). **(D)** Gene expression levels of *KLF4, Atf4, Chop, Beclin1, Atg5, IL-18*, and *IL-1β* in adipocytes infected with pAd-*KLF4* or si-*KLF4* and incubated with leptin or not (*n* = 3). **(E)** Graphic representation of a network of the target genes. Bioinformatics analysis of the protein–protein interaction. **(F)** Atg5 interacted with Atf4. Coimmunoprecipitation (Co-IP) analysis was done in HA-Atg5 and His-Atf4 transfected HEK293 cells. **(G)** Co-IP analysis was done in pAd-*Atg5* and pAd-*Atf4* transfected adipocytes with no infection were used as control. Values are means ± SEM. *p < 0.05 compared with the TM group, ^#^*p* < 0.05 compared with the leptin treatment group.

Having confirmed that leptin inhibited ER stress-induced autophagy in adipocytes, we next sought to explore the regulation of leptin on ER stress. As shown in Figures 6D,E, overexpression of *leptin* downregulated autophagy and inflammation both in the control group and in the 4-PBA group. And as indicated cluster of transcription factors of multiply genes were altered with leptin treatment (Figure 1B). In order to analyze the underlying mechanisms of leptin on ER stress, we firstly considered transcriptional-level control. Our results showed leptin increased the expression of *KLF4*, but TM treatment had the opposite effect (Figure 7A). Further analysis demonstrated the *Atf4* promoter region contained three potential binding domains of *KLF4* (Figure 7B). And measurements revealed that the binding site, 560–130 bp upstream of the initiation site of *Atf4* functioned (Figures 7B,C). We next treated adipocytes with the overexpression recombinant adenovirus vector of *KLF4* (pAd-*KLF4*) or interference recombinant lentiviral vector of *KLF4* (si-*KLF4*), in the presence of leptin or not. Figure 7D showed cotreatment of leptin and pAd-*KLF4* reduced the mRNA levels of *Atf4* and *Chop* significantly (Figure 7D). The data also indicated autophagy indicators *Beclin1* and *Atg5* were decreased with the overexpression of *KLF4*, and leptin addition further strengthened this trend (Figure 7D). Thus, these findings suggested *KLF4* inhibited the transcription of *Atf4*, and leptin enhanced the inhibition effect of *KLF4* and reduced ER stress of adipocytes.

### Atf4-Atg5 Complex Regulates Leptin-Mediated Autophagy in Mice Adipocytes

The transcriptional regulation of *Atf4* by *KLF4* led us to hypothesize whether ER stress affected adipocytes autophagy through a transcription regulation either. However, we failed to show this regulation network (data were not shown). The increased protein levels of autophagy-related proteins caused by TM treatment prompted us to further hypothesize that ER stress regulated autophagy by direct modification, through a physical interaction. Firstly based on the bioinformatics analysis and previous data sheet, we showed Atf4 interacted with Atg5 (Figure 7E). Then by protein–protein measurement, Atf4 protein interacted strongly with transfected Atg5 in both HEK293 cells and in adipocytes (Figures 7F,G). Thus, these data suggest that Atf4 and Atg5 directly bind, and then regulated autophagy progress in adipocytes.

**Figure 8:**
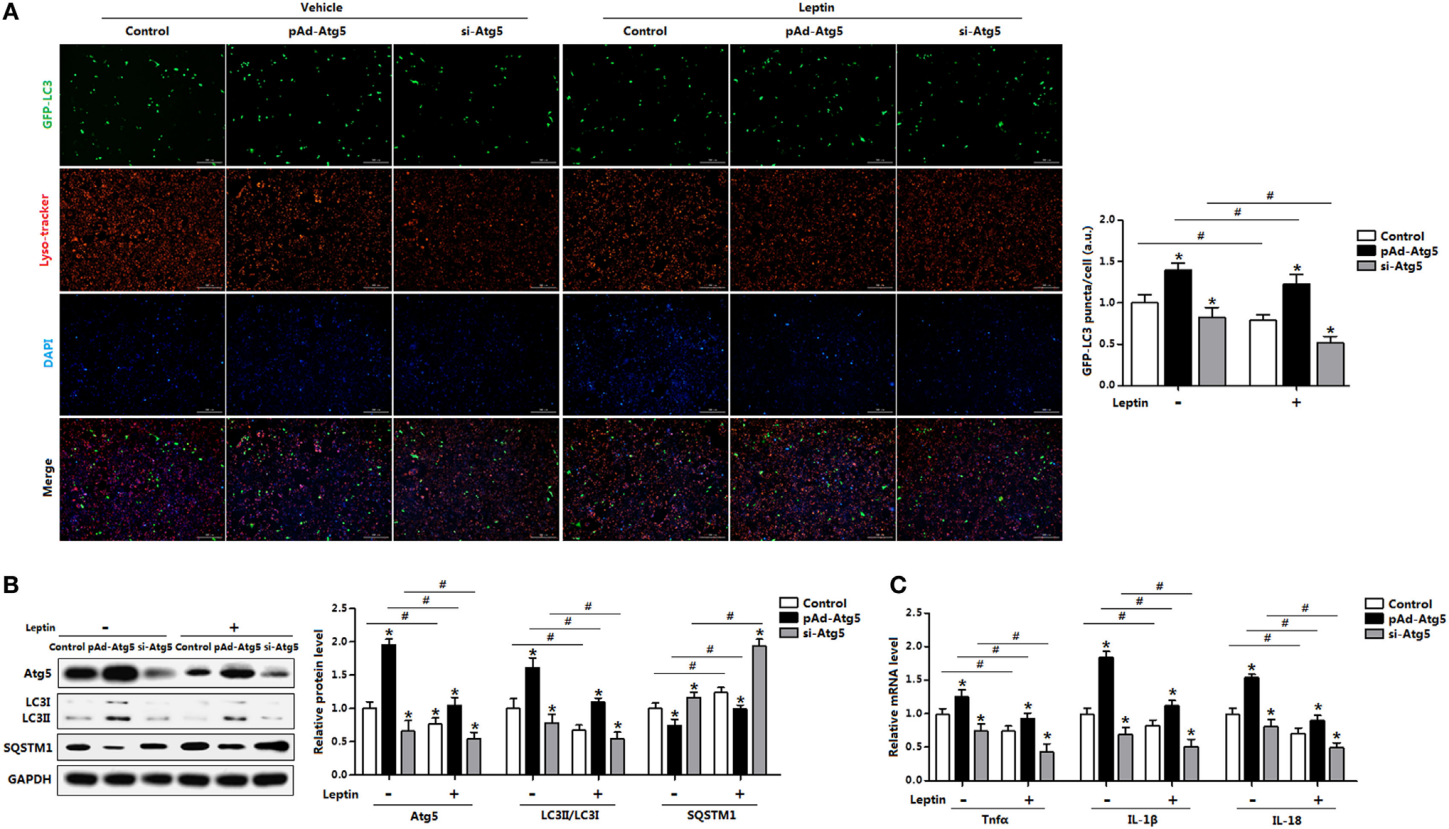
Activating transcription factor 4 (Atf4) forms a complex with Atg5 and drives leptin-mediates autophagy in mice adipocytes. Adipocytes were preinfected with pAd-*Atg5* or si-*Atg5* and incubated with tunicamycin (TM), followed treated with or without leptin (*n* = 3). **(A)** Representative fluorescent photomicrographs showing the GFP-LC3 puncta formation in adipocytes transfected with GFP-LC3 plasmid and stained with Lyso-tracker Red. The nuclei were stained with DAPI shown in blue. **(B)** Representative Western blots showing the protein levels of Atg5, LC3II, and SQSTM1. **(C)** Gene expression of *Tnfα, IL-1β*, and *IL-18* in adipocytes. Values are means ± SEM. *p < 0.05 compared with the TM group, ^#^*p* < 0.05 compared with the leptin group.

To determine whether Atg5 is responsible for leptin-mediated autophagy, we next employed a genetic approach, overexpression recombinant adenovirus vector of *Atg5* (pAd-*Atg5*) or interference recombinant lentiviral vector of *Atg5* (si-*Atg5*), to explore the underline mechanisms. As expected, interference of *Atg5* inhibited autophagy of adipocytes and the addition of leptin further reduced the punctate GFP-LC3 formation (Figure 8A). Consistently, LC3II protein level was downregulated in adipocytes cotreated with leptin and si-*Atg5* compared with that in the si-*Atg5* alone group (Figure 8B). Similar to the 3-MA blockage, gene-silencing of *Atg5* combine with leptin treatment also significantly inhibited the increasing of proinflammatory cytokines in adipocytes (Figure 8C). Overall, these findings implicated Atf4-Atg5 complex played key role in the process of leptin-mediated autophagy and inflammation in mice adipocytes.

### Atg5 Activates IκB Signaling Pathway in Autophagy-Mediated Adipocyte Inflammation

**Figure 9:**
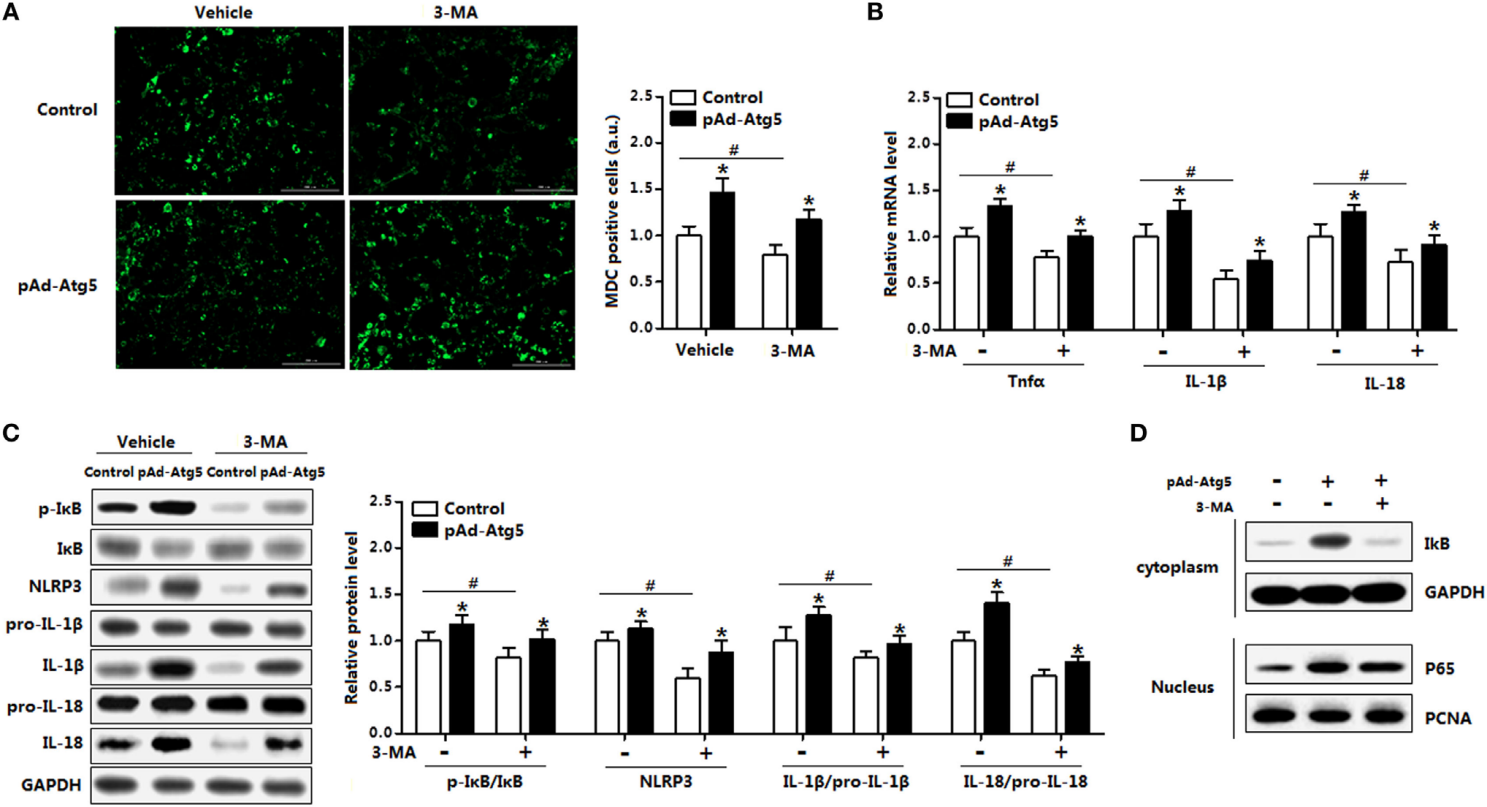
Autophagy-related gene 5 (Atg5) activates IκB signaling pathway in autophagy-mediated adipocyte inflammation. Adipocytes were infected with pAd-*Atg5* and incubated with tunicamycin (TM), followed with or without 3-methyladenine (3-MA) treatment (*n* = 3). **(A)** Representative pictures of autophagosome formation monitored by MDC staining. **(B)** Gene expression of *Tnfα, IL-18*, and *IL-1β*. **(C)** Protein level of phosphorylation IκB, NLRP3, IL-1β, and IL-18. **(D)** IκB and GAPDH protein levels in cytoplasm and P65 and PCNA protein levels in nuclear extracts of the adipocytes. Full scans of uncropped blots are included in Figure S3. Values are means ± SEM. *p < 0.05 compared with the TM group, ^#^*p* < 0.05 compared with the 3-MA treatment group.

Having confirmed that Atg5 functioned in leptin-mediated autophagy and inflammation, we then sought to examine the underlying regulation mechanism of Atg5 in adipocytes inflammation. Overexpression of *Atg5* significantly increased autophagy of adipocytes that preincubated with 3-MA (Figure 9A); it also enhanced inflammation by elevating the expression of *Tnfα, IL-1β*, and *IL-18* which were inhibited with 3-MA incubation (Figure 9B). We then measured the protein levels of phosphorylation IκB, NLR family, pyrin domain containing 3 (NLRP3), IL-18, and IL-1β. Results showed the inflammatory indicators such as p-IκB, NLRP3, and IL-1β were all increased with pAd-*Atg5* alone treatment, and still increased when cells were cotreated with *Atg5* and 3-MA (Figure 9C). We then investigated whether the effect of Atg5 on inflammation was correlated with the degradation of IκB protein. Forced expression of *Atg5* greatly increased the protein level of IκB in the cytoplasm and the protein level of p65 in the nuclear extracts (Figure 9D). And this also happened when cells were pretreated with 3-MA (Figure 9D). These findings thus initiated underscore that Atg5-mediated autophagy attenuated inflammation *via* IκB signaling pathway.

### Leptin Administration Reduces Autophagy and Inflammation of Adipose Tissue *In Vivo*

**Figure 10:**
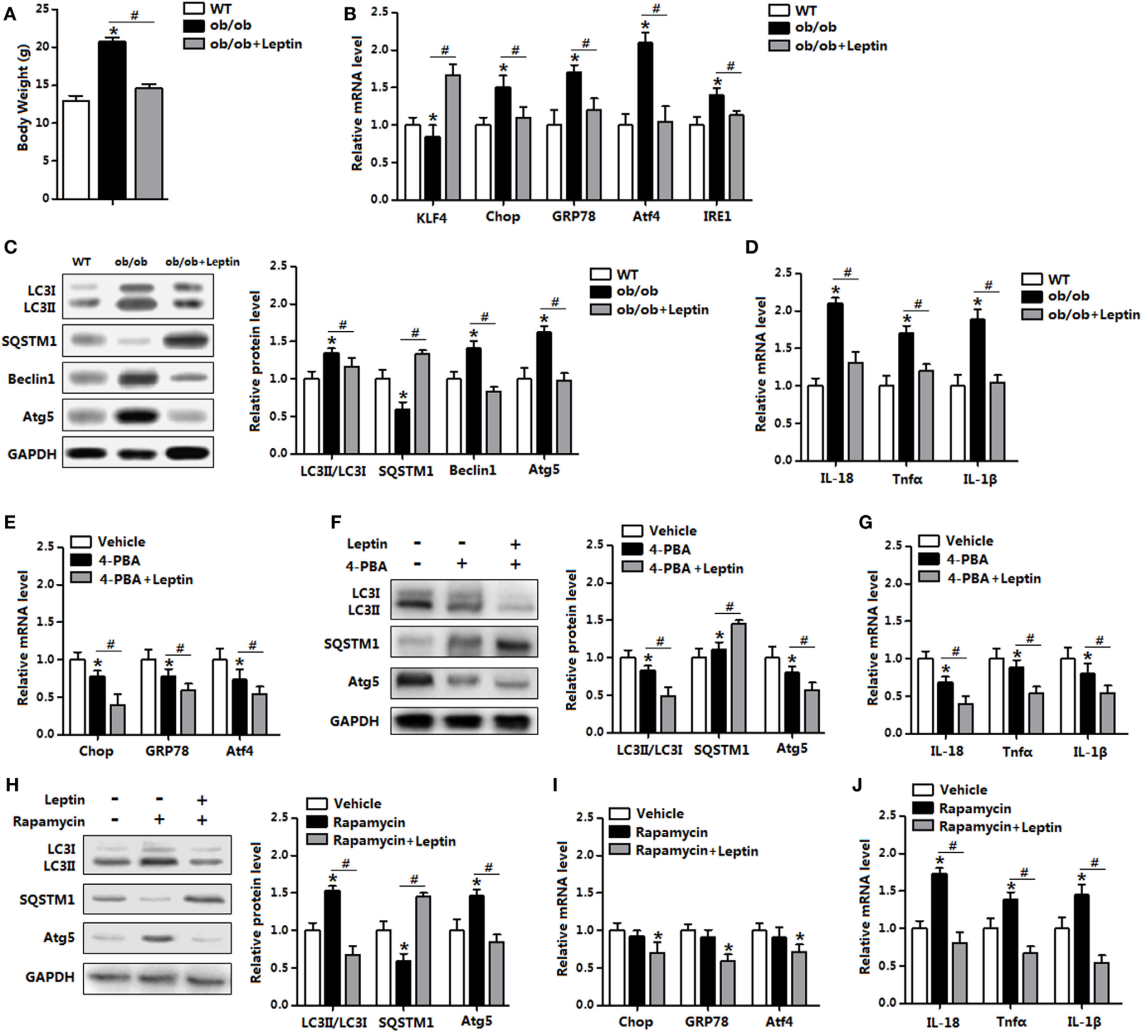
Leptin administration reduced autophagy and inflammation of adipose tissue *in vivo*. From **(A–D)** wild-type (WT) mice and *ob/ob* mice were injected with tunicamycin ™, and followed treated with recombinant leptin protein or not (*n* = 6). **(A)** Body weight of the mice. **(B)** Gene expression level of Krüppel-like factor 4 (*KLF4*), C/EBP homologous protein (*Chop*), glucose-regulated protein 78 (*GRP78*), activating transcription factor 4 (*Atf4*), and inositol-requiring enzyme 1 (*IRE1*) in the mice white epididymal adipose tissue (eWAT). **(C)** The conversion of LC3I to LC3II and protein levels of Atg5, Beclin1, and SQSTM1 were examined by Western blot in the eWAT of mice. **(D)** Gene expression level of *IL-18, Tnfα*, and *IL-1β* in mice eWAT. From **(E–G)** WT mice were pretreated with TM and 4-phenylbutyric acid (4-PBA), and followed treated with recombinant leptin protein or not (*n* = 6). **(E)** Gene expression of ER stress markers in the eWAT of mice. **(F)** Representative Western blots showing the protein levels of Atg5, LC3II, and SQSTM1 in the eWAT of mice. **(G)** Gene expression of *Tnfα, IL-18*, and *IL-1β* in the eWAT of mice. From **(H–J)** WT mice were pretreated with TM and rapamyclin, and followed treated with recombinant leptin protein or not (*n* = 6). **(H)** Representative Western blots showing the protein levels of Atg5, LC3II, and SQSTM1 in the eWAT of mice. **(I)** Gene expression of ER stress markers in the eWAT of mice. **(J)** Gene expression of *Tnfα, IL-18*, and *IL-1β* in the eWAT of mice. Full scans of uncropped blots are included in Figure S3. Values are means ± SEM. *p < 0.05 compared with the control group, ^#^*p* < 0.05 compared with the leptin group.

To further explore the *in vivo* relevance of our findings, we next determined the induction of ER stress and autophagy in mice treated with leptin. As shown in Figure 10A, leptin injection significantly reduced body weight in leptin genetic deficiency mice (*ob/ob* mice). The replenish of leptin induced a markedly decrease in the levels of ER stress markers, while increased the level of *KLF4* (Figure 10B). Consistently, autophagic marker proteins LC3II, Beclin1, and Atg5 were all reduced with leptin injection compared with those in the *ob/ob* mice (Figure 10C). As expected, the gene expressions of pro-inflammatory cytokines were also reduced with leptin injection (Figure 10D).

Next, we used leptin administration in the WT mice which had been trigged ER stress by TM injection. Leptin treatment further reduced ER stress in 4-PBA pretreated mice (Figure 10E). In addition, 4-PBA administration decreased the protein levels of LC3II and Atg5, but increased SQSTM1 level. The addition of leptin further strengthened the autophagy blockage and similarly reduced adipose inflammation (Figures 10F,G). As shown in Figures 10H,I, leptin treatment significantly reduced rapamycin-induced autophagy, along with the downregulation of ER stress. Additionally, the addition of leptin inhibited adipose inflammation in rapamycin injected mice (Figure 10J). Thus, these data verify the role of leptin in the administration of ER stress and autophagy *in vivo*.

## Discussion

Autophagy is an essential lysosome-mediated bulk degradation pathway for cellular survival, development and homeostasis (17, 42). Particularly, autophagy is essential for by endolysosomal degradation and elimination of misfolded proteins and damaged organelles during ER stress (43, 44). Although recent investigations have revealed that ER stress can either stimulate or inhibit autophagy in different cell types, it still does not determine crosstalk between ER stress and autophagy in adipocytes (43, 45–48). Meanwhile, the ER stress-associated molecular cues that control the switch of autophagy are also obscure. In this study, we demonstrated that exogenous TM and Tg stimulated ER stress and autophagy in adipose tissue. These findings are consistent with our previous and other studies that ER stress is sufficient to trigger autophagy and reduce adiponectin expression in adipocytes (12, 49, 50). Moreover, ER stress also initiated inflammation but decreased leptin level in adipocytes. Interestingly, autophagy-deficient adipose tissue has drastically reduced leptin secretion (51). We then investigated the expression profile of core autophagy and inflammation genes in leptin-treated or TM-treated adipose tissue. Our data showed TM elevated but leptin reduced the autophagy-related genes such as *Beclin1, Atg5*, and *Atg12*. Consistently, kinds of biological process and pathways correlated with cellular inflammation response were enriched in the GO analysis. Furthermore, we uncovered an unexpected result that leptin inhibited ER stress-induced autophagy and inflammation of adipocyte, though the status of inflammation and autophagy were both elevated in TM-induced ER stress. Previous studies show that ER stress induces adipocyte inflammation and insulin resistance by activating NF-κB pathways (52, 53). Although several previous studies investigate the link between ER stress and autophagy, it remains unclear how leptin affects the crosstalk between ER stress and autophagy (54, 55). It is interesting to note that leptin might be an upstream regulator and be essential for inhibiting ER stress-induced autophagy and alleviating adipocyte inflammation in downstream.

Based on these findings, we first explored the regulatory role of leptin on adipocyte autophagy. Leptin is a well-known adipocyte-derived hormone involved in food intake and energy metabolism (1, 56). Several reports have implicated increased accumulation of autophagosomes possibly functions as a pathogenic signal contributing to induction of ER stress-mediated inflammation (57, 58). In this study, we preliminarily determined that leptin inhibited ER stress-induced autophagy in adipocytes and adipose tissue with downregulating the autophagic flux by LC3II turnover, SQSTM1 degradation and reduced autophagosome clearance. These findings are consistent with other studies that leptin inhibits canonical autophagy in peripheral tissues including skeletal muscle, heart and liver by reducing LC3II, Beclin1, and Atg5 (59, 60). Accordingly, we also employed the approaches of pharmacological inhibition which impeded either early autophagosome formation (by 3-MA) or late autolysosome formation (by BafA1) phases of autophagy signaling, or the ER stress inhibitor (4-PBA) to specifically determine the roles of ER stress and autophagy in mediating the effects of leptin on adipocyte inflammation. Our findings demonstrated that autophagy inhibitor, 3-MA further accelerated leptin-reduced autophagy in adipocytes, while still maintaining leptin-mediated downregulation of ER stress. These findings thus suggested that leptin-mediated inhibition of ER stress was the upstream of autophagy response, which ultimately resulted in the reduction of inflammation as evidenced by decreasing the proinflammatory cytokines expression. Moreover, Saroj et al. find that leptin induces autophagy and promotes apoptosis in cancer cells (61). Indeed, we speculate that cellular events, i.e., autophagy, ER stress and apoptosis, respond to leptin very differently in normal and cancer cells (62, 63). Similar to these findings, it is thought that ER stress initiates autophagy only when aggregated proteins become excessive enough to overwhelm the canonical ubiquitin-proteasome-dependent ER-associated degradation (64, 65). Moreover, studies indicate the transcriptional upregulation of LC3 and Atg5 depends on Atf4 and Chop induction during ER stress-induced autophagy (66, 67). Intriguingly, our present study found that Atf4 directly interacted with Atg5 and the complex was formed to mediate autophagy and inflammatory response in adipocytes. Furthermore, we showed that *KLF4* bound to the *Atf4* promoter region then inhibited the transcription of *Atf4*, suggesting that leptin inhibited ER stress of adipocytes through activating JAK2/STAT3 pathway and promoting *KLF4* transcriptional inhibition of *Atf4*. Recent studies also confirm that KLF4 mediates leptin’s effects in hypothalamic arcuate nucleus (68, 69). Another intriguing observation from the present study was that Atf4 had a protein–protein interaction with Atg5 and subsequently initiated adipocyte autophagy. The main events of autophagy, such as phagophore formation and maturation, are substantially maintained by the ATG12-ATG5 conjugate (70–72). This observation was further confirmed by the results that inhibition of Atg5 abolished leptin signaling-mediated autophagy induction in adipocytes. Collectively, our findings provide the first evidence that leptin signal regulates autophagy of adipocytes through the Atf4-Atg5 signal, although how leptin regulate protein activity of Atf4-Atg5 complex still requires further investigation.

One question remains to be answered is that how leptin inhibited ER stress-induced inflammation by activating autophagy. Increasing evidence demonstrates the importance of NLRP3 inflammasome in the regulation of adipocyte inflammation (73, 74). IκB kinase is one of the most important kinases that mediates the effects of general inflammatory stimuli inside adipocyte and is a major upstream regulator of NLRP3 inflammasome (8, 75, 76). In the current study, we demonstrated that leptin reduced NLRP3 protein level and inflammatory factors expression by inhibiting Atg5-mediated autophagy. These findings are consistent with the studies proposed the essential relationship between autophagosomes formation and NLRP3 inflammasome activation (77, 78). Unexpectedly, our result also showed that reduction of autophagy by 3-MA ameliorated inflammation as leptin functioned in adipocyte. Though TM-induced ER stress can directly activate NLRP3 inflammasome and mediate p65 translocation in nucleus which has been confirmed by our data and other studies, further investigations are needed to resolve the detail molecular mechanisms (79, 80).

**Figure 11:**
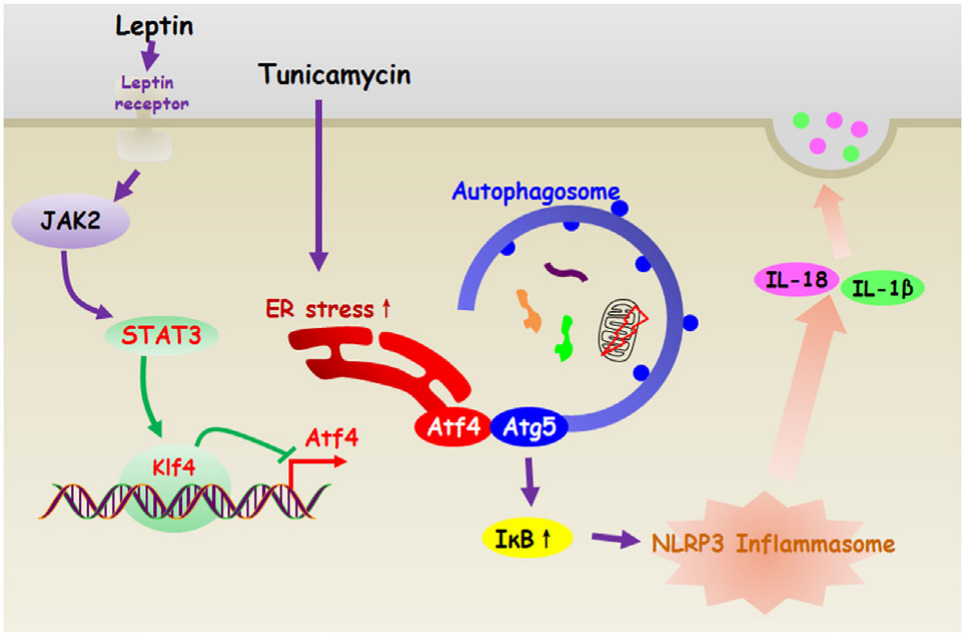
Leptin inhibited endoplasmic reticulum (ER) stress-mediated autophagy and inflammation through the negatively regulation of activating transcription factor 4 (Atf4)/Atg5 complex in adipocytes. And leptin is essential for transcriptional regulation of Atf4/Atg5 signal during the NLRP3 inflammasome degradation in adipocytes.

In summary, our present study demonstrates that leptin inhibits ER stress-induced inflammation through reducing Atf4-Atg5-mediated autophagy in adipocytes. Moreover, leptin is essential for transcriptional regulation of Atf4/Atg5 signal during the NLRP3 inflammasome degradation in adipocytes (Figure 11). Thus, our results provide a novel therapeutic option for exploring the proteasome or autophagy activator to reverse obesity-related metabolic disorders.

## Data Accession

The raw data have been deposited to NCBI Sequence Read Archive (SRA). The NCBI SRA accession: SRP119756.

## Ethics Statement

Mice handling protocols were conducted following the guidelines and regulations approved by the Animal Ethics Committee of Northwest A&F University.

## Author Contributions

LG and CS designed research. All authors performed research. ZL and DL analyzed data. QR, HW, and CL contributed reagents/analytic tools. LG and ZL wrote the article.

## Conflict of Interest Statement

The authors declare that the research was conducted in the absence of any commercial or financial relationships that could be construed as a potential conflict of interest.
