# Supplementary material for: Reduced Endoplasmic Reticulum Stress-Mediated Autophagy Is Required for Leptin Alleviating Inflammation in Adipose Tissue
Source: Front Immunol. 2017 Nov 8;8:1507. doi: 10.3389/fimmu.2017.01507 (PMC5715390; doi:10.3389/fimmu.2017.01507)
Supplement: Supplementary file 1 [file image_1.pdf]

Supplementary Material

Reduced ER stress-mediated autophagy is required for leptin alleviating inflammation in adipose tissue

Lu Gan, Zhenjiang Liu, Dan Luo, Qian Ren, Hua Wu, Changxing Li, Chao Sun*

***Correspondence:** Corresponding Author: sunchao2775@163.com

1 Supplementary Figures and Tables

1. Supplementary Figures

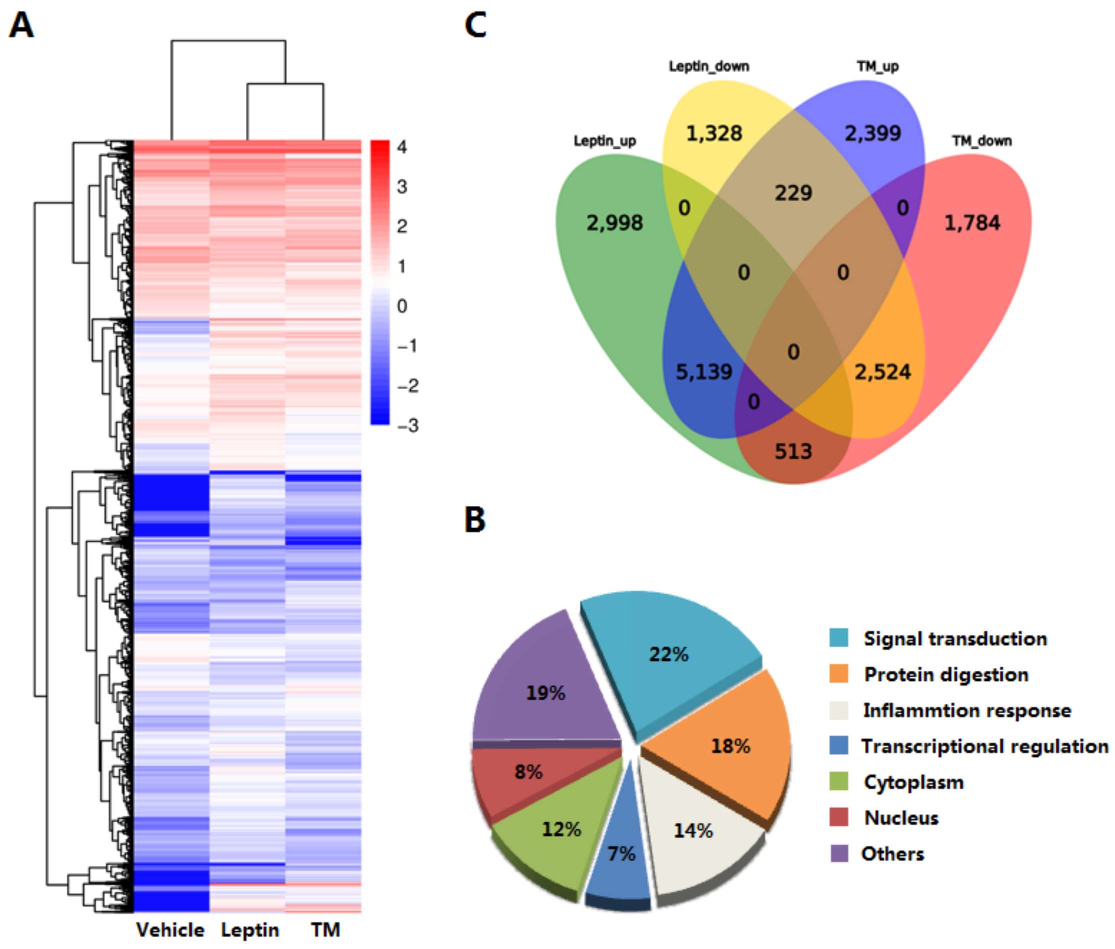

Supplementary Figure 1. Leptin treatment produces a transcriptional signature distinct from tunicamycin treatment in mice adipose (A) Heatmap showing the genes significantly up- or down-regulated in response to TM or leptin treatment (n=3). (B) Gene Ontology (GO) analysis of the altered genes in Figure 2A, which shows the response of the biology processes to TM or leptin treatment. The area of each pie slice represents the number of genes that belong to the indicated GO terms (n=3). (C) Venn diagrams depicting the distinct and intersecting nature of gene sets whose transcription is significantly regulated by treatment with TM or leptin as compared to baseline vehicle control (n=3).

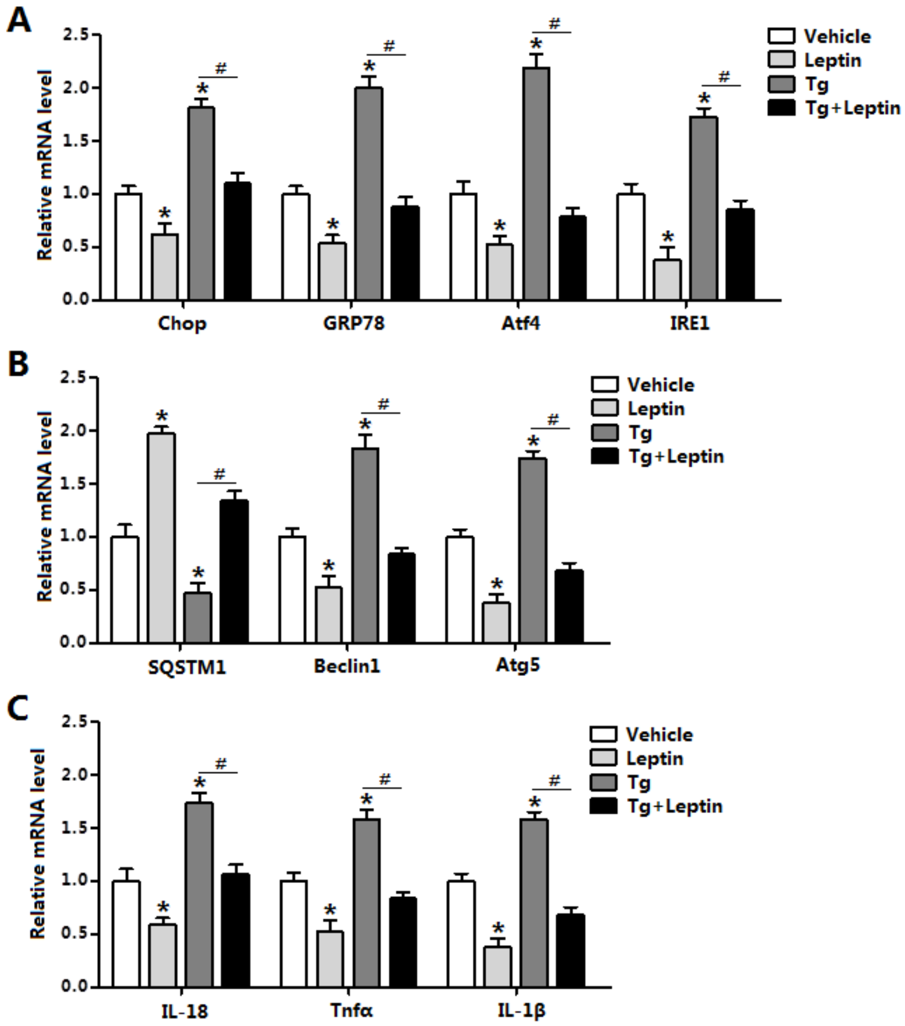

Supplementary Figure 2. Leptin alleviated thapsigargin (Tg)-induced ER stress, autophagy and inflammation in mice adipose tissue. (A) Changes in the expression level of genes associated with ER stress (n=3). (B) Changes in the expression level of genes associated with autophagy (n=3). (C) Changes in the expression level of genes associated with inflammation (n=3). Values are means ± SEM. * $p < 0.05$ compared with the vehicle group, $^{\#}$ $p < 0.05$ compared with the Tg group.

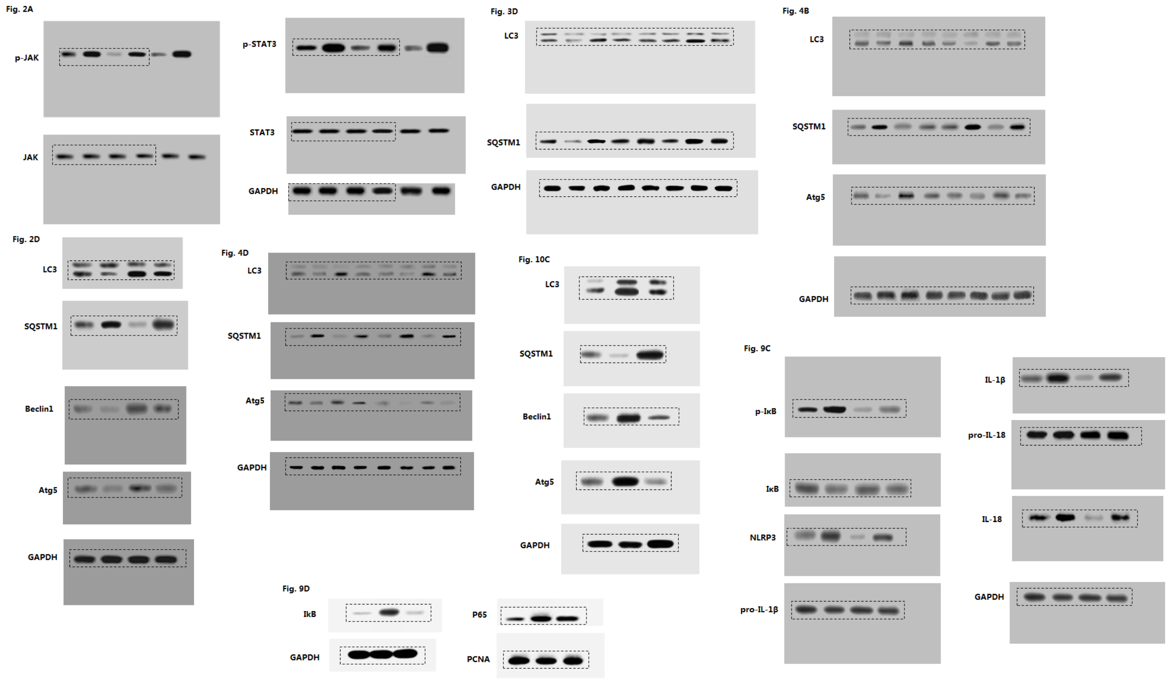

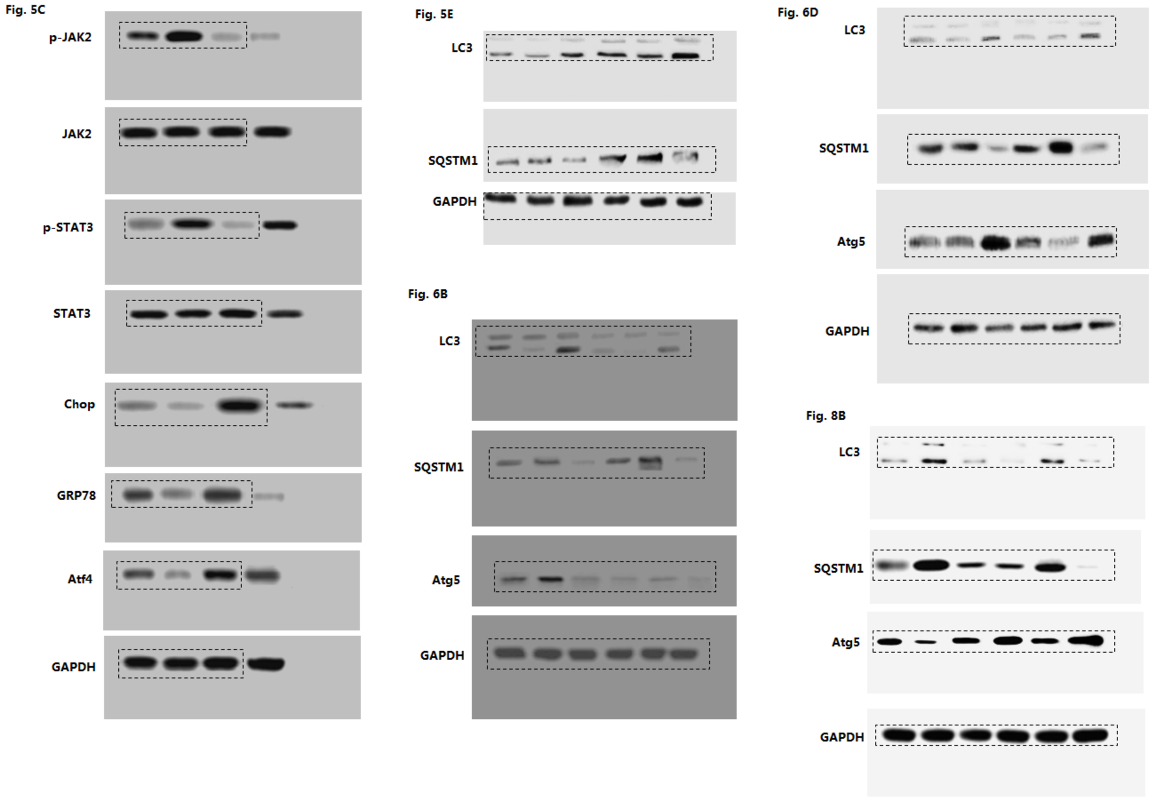

Supplementary Figure 3. The original full gels and blots related with the Figure 2-Figure 10.
